# Vision and Language Reference for a Segment Anything Model for Few-Shot Segmentation

**DOI:** 10.3390/jimaging12040143

**Published:** 2026-03-24

**Authors:** Kosuke Sakurai, Ryotaro Shimizu, Masayuki Goto

**Affiliations:** 1 Graduate School of Creative Science and Engineering, Waseda University, Tokyo 169-8555, Japan; 2 Institute of Data Science, Waseda University, Tokyo 169-8555, Japan; shi3mizu8-r@fuji.waseda.jp; 3 School of Creative Science and Engineering, Waseda University, Tokyo 169-8555, Japan; masagoto@waseda.jp

**Keywords:** image segmentation, few-shot segmentation, segment anything model, vision-language model, multimodal learning

## Abstract

Segment Anything Model (SAM)-based few-shot segmentation models traditionally rely solely on annotated reference images as prompts, which inherently limits their accuracy due to an over-reliance on visual cues and a lack of semantic context. This reliance leads to incorrect segmentation, where visually similar objects from different categories are incorrectly identified as the target object. We propose Vision and Language Reference Prompt into SAM (VLP-SAM), a novel few-shot segmentation model that integrates both visual information of reference images and semantic information of text labels into SAM. VLP-SAM introduces a vision-language model (VLM) with pixel–text matching into the prompt encoder for SAM, effectively leveraging textual semantic consistency while preserving SAM’s extensive segmentation knowledge. By incorporating task-specific structures such as an attention mask, our model achieves superior few-shot segmentation performance with only 1.4 M learnable parameters. Evaluations on PASCAL-5^i^ and COCO-20^i^ datasets demonstrate that VLP-SAM significantly outperforms previous methods by 6.8% and 9.3% in mIoU, respectively. Furthermore, VLP-SAM exhibits strong generalization across unseen objects and cross-domain scenarios, highlighting the robustness provided by textual semantic guidance. This study offers an effective and scalable framework for few-shot segmentation with multimodal prompts.

## 1. Introduction

**Figure 1:**
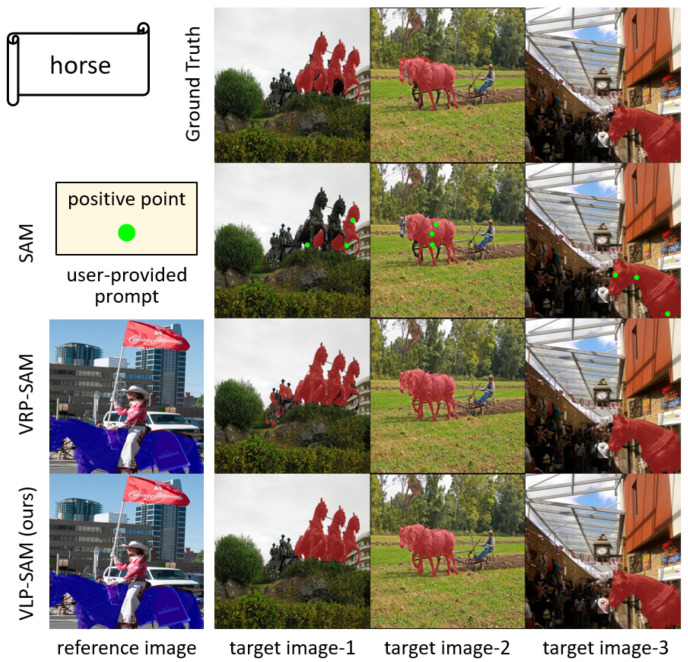
Visualization results of our proposed method VLP-SAM compared to SAM [1] and VRP-SAM [2]. VLP-SAM segments target objects by inputting a reference image and a text label (e.g., “horse”) instead of user-provided prompts.

In recent years, the Segment Anything Model (SAM) [1] has emerged as a foundational image segmentation model trained on a large dataset with a billion mask labels. SAM has powerful zero-shot capabilities that can segment any object in a target image by taking user-provided prompts consisting of points, bounding boxes, or coarse masks. However, as shown in Figure 1, user-provided prompts require the user’s comprehensive understanding of the target objects, and different customized prompts are needed for each target image. Furthermore, SAM outputs category-agnostic masks, limiting its usability in real-world applications.

Few-shot segmentation models [2,3,4,5] addressed these issues by inputting a few annotated reference images as prompts to SAM and can segment specific objects in target images without user-provided prompts. Based on the pixel-level similarity between annotated reference images and target images, few-shot segmentation models segment target objects of the same category as the reference images. On the other hand, previous SAM-based few-shot segmentation models only use annotated reference images as prompts and lack semantic information. Importantly, relying purely on visual similarity causes a “semantic gap,” where the model fails when segmenting objects that are visually similar but semantically different.

In this work, we propose a novel few-shot segmentation model, Vision and Language Reference Prompt into SAM (VLP-SAM), that inputs not only reference images but also text labels as prompts to SAM. By using text information, VLP-SAM can leverage not only the visual similarity between annotated reference images and target images but also the semantic similarity between text labels and target objects. For example, as shown in Figure 1, the previous few-shot segmentation model VRP-SAM [2] segments the different objects, such as a human figure visually similar to the horse in target image-1, because it relies solely on the visual similarity of the reference image. On the other hand, VLP-SAM captures the semantic similarity by inputting the text label, and accurately segments only “horse”.

In particular, VLP-SAM introduces an efficient and scalable architecture that trains a new prompt encoder for SAM, termed the VLP encoder, with only 1.4 M learnable parameters. This encoder integrates a multimodal vision-language model (VLM) to generate prompt embeddings from a target image, an annotated reference image, and a text label. Specifically, we employ a VLM with pixel–text matching, such as CLIP Surgery [6], rather than standard CLIP [7], which is trained solely on global CLS tokens. This approach facilitates the fine-grained, pixel-level localization required for precise segmentation. To further refine the semantic information of target objects, the VLP encoder incorporates two novel components: the textual prototype and the attention mask. Specifically, the textual prototype provides global semantic guidance by injecting class-specific features to identify what the target object is, while the attention mask acts as a semantic spatial mask providing local guidance to highlight where class-relevant regions are located. These components extract high-level semantic guidance from the text label, thereby significantly boosting accuracy even with minimal learnable parameters. Finally, the prompt embeddings created by the VLP encoder are input to the SAM’s mask decoder, allowing the VLP-SAM to segment target objects without user-provided prompts.

To demonstrate the effectiveness of VLP-SAM, we conducted experiments on the PASCAL-$5^{i}$ [8] and COCO-$20^{i}$ [9] datasets. Our experimental results show that VLP-SAM significantly outperforms the previous model by a large margin (6.8% and 9.3% in mIoU, respectively) in a few-shot segmentation task. Furthermore, VLP-SAM exhibits superior generalization capability across unseen objects and cross-domain scenarios, and its effectiveness is further validated through qualitative visualizations and extensive ablation studies.

The main contributions are summarized as follows:

- We propose Vision and Language Reference Prompt into SAM (VLP-SAM), which is the first SAM-based few-shot segmentation model to integrate both textual labels and visual reference images.
- We design an innovative prompt encoder, VLP encoder, that integrates a pixel–text matching VLM to incorporate textual prototypes and attention masks with minimal learnable parameters.
- We demonstrate that VLP-SAM outperforms previous models by a large margin in unseen objects and cross-domain scenarios. Furthermore, we validate the effectiveness of our approach through qualitative visualizations and extensive ablation studies.

## 2. Related Work

This paper proposes a new few-shot segmentation model, VLP-SAM, which inputs not only reference images but also text labels as prompts to SAM. First, this section summarizes the relevant research on SAM and its extended models. Next, regarding SAM-based few-shot segmentation models, since various studies have already been reported, an overview of the entire body of prior studies in this field is described. Finally, we provide an overview of the multimodal visual-language models (VLMs) introduced in this research and summarize related studies.

### 2.1. Segment Anything Model

The Segment Anything Model (SAM) [1] is a category-agnostic interactive segmentation model trained on a large-scale SA-1B dataset containing over 1 billion masks. SAM shows powerful zero-shot capabilities for new objects without additional training by taking user-provided prompts consisting of points, bounding boxes, or coarse masks. Due to its generality, SAM has been applied in diverse research areas [10,11,12,13,14,15,16,17]. In particular, recent segmentation models [18,19,20,21] have also advanced the general-purpose segmentation field and further broadened the context of our work.

**Figure 2:**
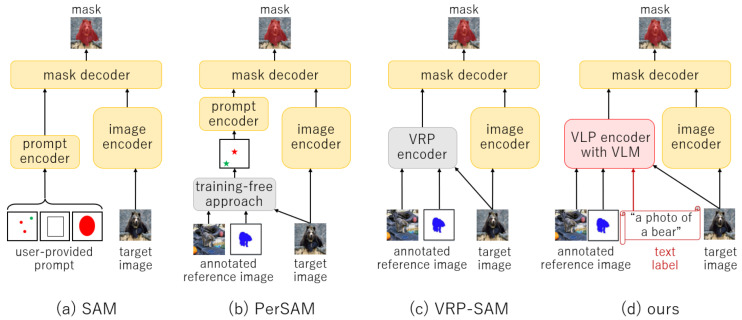
Comparison of our proposed method, VLP-SAM, and three previous models. (**a**) SAM [1] is a category-agnostic interactive zero-shot segmentation model that inputs user-provided prompts for each target image. (**b**) PerSAM [3] is a training-free SAM-based few-shot segmentation model that inputs positive and negative points on the target image into SAM, without requiring user-provided prompts. (**c**) VRP-SAM [2] is a SAM-based few-shot segmentation model incorporating a VRP encoder to generate prompt embeddings for SAM from an annotated reference image. (**d**) Our proposed method, VLP-SAM, introduces a novel prompt encoder for SAM, called the VLP encoder, which accepts both reference images and text labels as input via a vision-language model.

As depicted in Figure 2a, SAM comprises three modules: an image encoder, a prompt encoder, and a mask decoder. The image encoder is a Vision Transformer [22] backbone to extract image embeddings. The prompt encoder generates prompt embeddings from geometric prompts such as points and boxes. The mask decoder is a Transformer-based decoder [23] that outputs a class-agnostic mask from image embeddings and prompt embeddings. While SAM can segment any object in zero-shot, it requires user-provided prompts for each target image, meaning human interaction and knowledge of the target image are needed. Furthermore, SAM does not output the class labels for each mask, limiting its usability in real-world applications.

### 2.2. Few-Shot Segmentation Model with SAM

Few-shot segmentation models [24,25,26,27,28,29,30,31,32] aim to segment objects in target images belonging to the same category as annotated reference images. Specifically, SAM-based few-shot segmentation models [2,3,4,5,33,34,35,36,37,38] only take a few reference image–mask pairs as prompts, instead of user-provided prompts for each target image. This allows leveraging the richness of a large-scale foundational model, SAM, while addressing SAM’s weaknesses: user interaction and class-agnostic masks.

SAM-based few-shot segmentation models can be mainly classified into two types: training-free models [3,4,35,36,37,39] that input geometric prompts derived from annotated reference images into SAM’s prompt encoder, and meta-learning models [2,5,38] that introduce the new SAM’s prompt encoder and input prompt embeddings into SAM’s mask decoder. Training-free models generate geometric prompts of target objects from pixel-level correlations between annotated reference images and target images without user interaction. For example, as shown in Figure 2b, PerSAM [3] selects the most positive and negative points from pixel-level correlations to input into SAM as prompts, and segments target objects without training. However, the performance of training-free models heavily depends on the quality of pseudo masks generated from pixel-level correlations, leading to incorrect prompts and reduced accuracy. Additionally, training-free models limit their scalability because SAM only accepts points, boxes, and masks as prompts.

Meta-learning models generate prompt embeddings for SAM derived from annotated reference images instead of geometric prompts via a meta-learning procedure. As depicted in Figure 2c, VRP-SAM [2] introduces a novel prompt encoder, the VRP encoder, which outputs prompt embeddings from annotated reference and target images. These prompt embeddings are then input into SAM’s mask decoder, creating a scalable few-shot segmentation model capable of predicting unseen classes not included in the training data. In this study, as shown in Figure 2d, we introduce a novel SAM’s prompt encoder (VLP encoder) that leverages a vision-language model (VLM) to capture both visual and semantic information for higher segmentation accuracy.

### 2.3. Multimodal Vision-Language Model

Multimodal vision-language models (VLM) embed images and text in a unified embedding space, and various VLMs have been released [7,40,41,42,43]. A representative model, CLIP [7], is a foundational model learned through contrastive learning [44,45] on 400 million image–text pairs. Its zero-shot capability has been widely utilized in various computer vision tasks [46,47,48,49,50,51,52]. However, for object detection and image segmentation tasks, pixel–text matching rather than CLS token-text matching, as seen in CLIP, is required to capture object-level features. VLM with pixel–text matching [6,53,54,55] can capture spatial and semantic information of objects from each patch–text relationship. Notably, CLIP Surgery [6] enhances the class attention map (CAM) [56,57,58,59] of CLIP to create high-performance pixel–text correlations (attention maps) without additional training. This model retains the benefits of CLIP’s large-scale foundational model while providing image–text embeddings that capture object-level semantic features. Therefore, in this study, we employ CLIP Surgery as VLM and CLIP as a comparison.

## 3. Methodology: VLP-SAM

In this paper, we propose Vision and Language Reference Prompt into SAM (VLP-SAM), a novel few-shot segmentation model that integrates both visual reference images and text labels as prompts for SAM. By leveraging text labels with VLM, VLP-SAM can utilize both the visual information of reference images and the semantic information of text labels.

**Figure 3:**
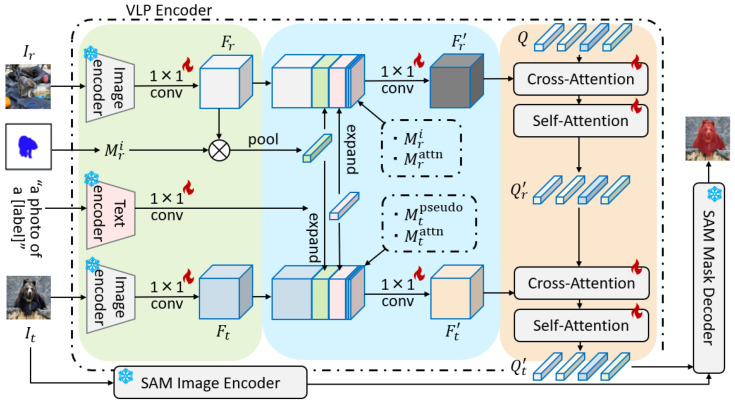
Overview of our proposed method, VLP-SAM. VLP-SAM introduces a novel prompt encoder for SAM, called the VLP encoder, which generates the prompt embedding $Q_{t}^{\prime }$ with vision-language information. In particular, the VLP encoder first embeds the target image $I_{t}$, the annotated reference image $I_{r}$, and the text label (“a photo of a [label]”) into the unified embedding space using the VLM encoder. These embeddings are then concatenated with prototypes and masks that aggregate information about the target object and the text label. Finally, the prompt embedding $Q_{t}^{\prime }$, refined through Transformer-based attention, is fed into SAM’s mask decoder, enabling VLP-SAM to produce a more accurate mask of the target object.

An overview of VLP-SAM is shown in Figure 3. A newly designed SAM’s prompt encoder, VLP encoder, generates prompt embeddings from the target image, the annotated reference image, and the text label with minimal learnable parameters. By inputting these prompt embeddings with vision-language information into SAM’s mask decoder, VLP-SAM outputs the mask of the target object. The details of the VLP encoder and the training process are described below.

### 3.1. VLP Encoder

In the few-shot segmentation task, the reference image $I_{r}$, its annotation mask $M_{r}^{i}$, where *i* denotes the category of the annotated object, and the target image $I_{t}$, are given. Additionally, this study prepares the class label of the annotated object *i* and uses it as a text $T_{i}$, such as “a photo of a [*i*]”. By utilizing the information from the annotated reference image $I_{r}+M_{r}^{i}$ and the text label $T_{i}$, VLP-SAM can accurately predict the mask $M_{t}^{i}$ of the category *i* in the target image.

First, using a multimodal VLM, $I_{r}$, $I_{t}$, and $T_{i}$ are embedded into the same latent space, and image embeddings $F_{r}\in R^{C\times H\times W}$, $F_{t}\in R^{C\times H\times W}$, text embedding $F_{\mathrm{text}}\in R^{C\times 1}$ are obtained via a 1×1 convolution layer [60]. Since image embeddings require patch-level detail, CLIP Surgery [6], which facilitates pixel–text matching, is used instead of standard CLIP [7]. To prevent overfitting of the VLP encoder, the VLM is frozen during the training phase. Next, the visual prototype $P_{i}$ that aggregates the features of the target object *i* from $F_{r}$ and $M_{r}^{i}$ and the textual prototype $F_{\mathrm{text}}$ that aggregates the semantic information of category *i* are extracted. The visual prototype $P_{i}$ is the average embedding of the target object from the reference image $F_{r}$, and it is formulated as follows:

(1) $$P_{i}=\mathrm{MaskAvgPool}\left(F_{r},M_{r}^{i}\right).$$

Furthermore, the masks of the reference and target images are utilized to enhance the task-specific information. The mask of the reference image uses the annotation mask $M_{r}^{i}$, while the mask of the target image uses a pseudo mask $M_{t}^{\mathrm{pseudo}}$, which is obtained through a common training-free approach. The pseudo mask $M_{t}^{\mathrm{pseudo}}\in R^{H\times W}$ is created by the cosine similarity correlations $S\in R^{(H\times W)\times \left(H\times W\right)}$ between the target image and the annotated reference image. Additionally, the attention masks $M_{r}^{\mathrm{attn}},M_{t}^{\mathrm{attn}}\in R^{H\times W}$ of the reference and target images are utilized. The attention masks are derived from the cosine similarity between the image and text embeddings, effectively capturing semantic and spatial guidance from the text label. Enhanced image embeddings $F_{r}^{\prime }\in R^{C\times H\times W}$, $F_{t}^{\prime }\in R^{C\times H\times W}$ are obtained by concatenating the visual prototype $P_{i}$, textual prototype $F_{\mathrm{text}}$, pseudo mask $M_{r}^{i}$, $M_{t}^{\mathrm{pseudo}}$, and attention mask $M_{r}^{\mathrm{attn}}$, $M_{t}^{\mathrm{attn}}$ with $F_{r}$ and $F_{t}$ through a 1×1 convolution layer to reduce the dimension. These enhanced embeddings $F_{r}^{\prime }$, $F_{t}^{\prime }$ are formulated as follows:

(2) $$\begin{array}{cc} F_{r}^{\prime } & =\mathrm{Conv}(\mathrm{concat}\left(F_{r},P_{i},F_{\mathrm{text}},M_{r}^{i},M_{r}^{\mathrm{attn}}\right)), \end{array}$$

(3) $$\begin{array}{cc} F_{t}^{\prime } & =\mathrm{Conv}(\mathrm{concat}\left(F_{t},P_{i},F_{\mathrm{text}},M_{t}^{\mathrm{pseudo}},M_{t}^{\mathrm{attn}}\right)). \end{array}$$

Finally, the VLP encoder generates prompt embeddings for SAM using enhanced image embeddings $F_{r}^{\prime }$, $F_{t}^{\prime }$ and learnable queries $Q\in R^{N\times C}$, where *N* denotes the number of tokens in the prompt embeddings. The learnable queries Q initially interact with the reference features $F_{r}^{\prime }$ through cross-attention and self-attention layers to acquire category-specific information. Subsequently, these queries interact with the target features $F_{t}^{\prime }$ to acquire foreground information in the target image. These processes are formulated as follows:

(4) $$\begin{array}{cc} Q_{r}^{\prime } & =\mathrm{SelfAttn}(\mathrm{CrossAttn}\left(Q,F_{r}^{\prime }\right)), \end{array}$$

(5) $$\begin{array}{cc} Q_{t}^{\prime } & =\mathrm{SelfAttn}(\mathrm{CrossAttn}\left(Q_{r}^{\prime },F_{t}^{\prime }\right)). \end{array}$$

The final $Q_{t}^{\prime }$ represents the prompt embeddings for SAM, which effectively integrate both visual and semantic information. In this process, the textual prototype provides global semantic context, while the attention mask serves as a semantic spatial mask to localize target objects, thereby bridging the semantic and spatial gap for precise segmentation.

### 3.2. Training

The prompt embeddings obtained from the VLP encoder are input to SAM’s mask decoder instead of geometric prompts. VLP-SAM can predict the mask $M_{t}^{i}$ for the category *i* of the target image from the mask decoder by utilizing reference images and text labels. We employ Binary Cross-Entropy (BCE) loss and Dice loss to train the VLP encoder. The loss of VLP-SAM is formulated as follows:

(6) $$\begin{array}{cc} \mathrm{Loss} & =\underset{\mathrm{BCE}\;\mathrm{Loss}\;}{\underset{︸}{-\frac{1}{n}\sum_{j=1}^{n}\left[y_{j}\log\left(p_{j}\right)+\left(1-y_{j}\right)\log\left(1-p_{j}\right)\right]}}\\ \; & +\underset{\mathrm{Dice}\;\mathrm{Loss}\;}{\underset{︸}{1-\frac{2\sum_{j=1}^{n}\left(p_{j}\cdot y_{j}\right)+1}{\sum_{j=1}^{n}\left(p_{j}+y_{j}\right)+1}}}, \end{array}$$

where *n* represents the total number of pixels, $y_{j}$ denotes the pixel *j* of the ground truth mask $M_{gt}^{i}$, and $p_{j}$ denotes the pixel *j* of the predicted mask $M_{t}^{i}$. During training, the parameters of SAM’s image encoder, mask encoder, and VLM encoder are frozen to prevent overfitting. By leveraging the weights of pretrained foundational models, VLP-SAM enables few-shot segmentation into any classes not included in the training data.

## 4. Experiment

### 4.1. Settings

To demonstrate the effectiveness of VLP-SAM, we conducted experiments on the PASCAL-$5^{i}$ [8] and COCO-$20^{i}$ [9] datasets following the few-shot setting [2,27,61]. We validate the generalization capability of VLP-SAM by dividing both datasets into four quarters: three quarters are used for training, and the remaining quarter is used for testing. PASCAL-$5^{i}$ consists of 20 classes (15 for training and 5 for testing), while COCO-$20^{i}$ consists of 80 classes (60 for training and 20 for testing), with no class overlap between training and testing. Detailed breakdowns of the testing categories for each fold are provided in Appendix A. In each fold, 1000 reference-target pairs are randomly sampled for testing.

In the VLP encoder, we use CLIP [7] (ViT-B/16 [22]) and CLIP Surgery [6] (ViT-B/16) as the pretrained VLM backbone. For the image encoder of SAM, we use ViT-H. We used the AdamW optimizer [62] with an initial learning rate of 1$\times 10^{-4}$, a batch size of 8 and 100 epochs for PASCAL-$5^{i}$, 50 epochs for COCO-$20^{i}$, and 50 learnable queries. In addition, VLP-SAM sets the input text to the VLM as “a photo of a [label]”. Following previous work [2,4,63], the evaluation metric is the mean intersection over union (mIoU).

### 4.2. Results

#### 4.2.1. Comparison with VRP-SAM

We compare VLP-SAM with the previous baseline, VRP-SAM [2]. VRP-SAM is a SAM-based few-shot segmentation model that introduces a novel prompt encoder (VRP encoder) without inputting text labels. In our experiments, we use ResNet-50 [64] pretrained on ImageNet [65], CLIP (ViT-B/16, ResNet-50), and CLIP Surgery (ViT-B/16) as backbones in the VRP encoder. For a fair comparison, all settings are kept identical to those used in VLP-SAM.

Table 1 and Table 2 show the performance of one-shot segmentation on PASCAL-$5^{i}$ and COCO-$20^{i}$. Our proposed VLP-SAM (CLIP Surgery) achieves mIoU scores of 77.0 on PASCAL-$5^{i}$ and 60.3 on COCO-$20^{i}$, outperforming the previous model, VRP-SAM (ResNet-50), by a large margin (6.8% and 9.3%, respectively). Compared with the text-free VRP-SAM (CLIP Surgery) with the same backbone, VLP-SAM (CLIP Surgery) achieved significant performance improvements (8.6% and 11.4%, respectively) by incorporating text information and task-specific structures.

Notably, VLP-SAM (CLIP), which excludes the attention mask, slightly underperforms VRP-SAM (ResNet-50) across both datasets, indicating that the simple integration of textual information via a general VLM is insufficient for performance enhancement. Instead, the superior accuracy of our model is driven by a strategic design that couples pixel–text matching VLM with task-specific structures like textual prototype and attention mask. This design effectively bridges the semantic and spatial gap, providing the fine-grained guidance required for accurate few-shot segmentation. In summary, these results demonstrate the high accuracy of VLP-SAM and its superior generalization capability to unseen classes with only 1.4 M learnable parameters. For a rigorous empirical evaluation, we provide a detailed statistical analysis, including standard deviations in Appendix B.

#### 4.2.2. Comparison with Other Few-Shot Methods

We further evaluate the effectiveness of VLP-SAM by comparing it with various few-shot segmentation models on COCO-$20^{i}$ dataset. These include CNN-based models such as CyCTR [61], BAM [27], and HDMNet [63], as well as SAM-driven frameworks such as PerSAM [3], Matcher [4] with DINOv2 [66], VRP-SAM, FCP [38], CMaP-SAM [39], and SAM2-based FS-SAM2 [67]. As shown in Table 3, VLP-SAM (CLIP Surgery) achieves 60.3 mIoU on COCO-$20^{i}$ without training on novel test classes, outperforming all methods. These results demonstrate that VLP-SAM achieves strong performance compared to recent representative few-shot segmentation models, underscoring the superior effectiveness of our multimodal guidance.

#### 4.2.3. Evaluation in Cross-Domain Scenarios

We evaluate the effectiveness of VLP-SAM in a cross-domain scenario, where a significant domain gap exists between the training and testing datasets. This gap stems from fundamental differences in image distributions: while PASCAL-5^i^ generally features large, centered objects in iconic views with relatively simple backgrounds, COCO-20^i^ presents a more challenging distribution characterized by cluttered backgrounds, varying object scales (including many small instances), and non-iconic shooting perspectives. Following prior work [2], we train the model on COCO-$20^{i}$ and test it on PASCAL-$5^{i}$ to assess the robustness of VLP-SAM under domain shift. To ensure no class overlap between training and testing, we construct a novel class split for PASCAL-$5^{i}$, as detailed in Appendix A.

The experimental results summarized in Table 4 show that VLP-SAM (CLIP Surgery) demonstrates superior performance in cross-domain settings, achieving an mIoU of 79.3 and outperforming the previous model. These results clearly demonstrate the strong generalization capability and effectiveness of VLP-SAM in cross-domain scenarios, highlighting the importance of textual guidance for robust performance.

#### 4.2.4. Ablation Study

##### Robustness to Textual Prompt Variations

Since VLP-SAM introduces a textual modality to guide the segmentation process, evaluating its robustness across diverse linguistic formulations is essential. Table 5 summarizes the performance of VLP-SAM (CLIP Surgery) in the 1-shot setting on PASCAL-$5^{i}$ using various prompt types. To explore the boundaries of semantic generalization, we defined eight variations: (b) synonyms, replacing the label with semantically equivalent terms (e.g., “shuttle” for “bus”); (c) hypernyms, using broader category labels (e.g., “large vehicle” for “bus”); (d) disambiguation, providing multiple candidate labels in a “a photo of a [Target] or a [Non-target]” format; (e) descriptive phrases, utilizing natural language descriptions (e.g., “a large road vehicle for carrying many passengers” for “bus”); (f) simple label, using only the category name as a prompt without the standard “a photo of a [label]” template (e.g., “[label]”); (g) label description, appending a descriptive phrase to the standard template (e.g., “a photo of a [label], [description]”); (h) typos, introducing character-level perturbations (e.g., “buss” for “bus”); and (i) incorrect label, which provides intentionally misleading categorical noise by replacing the label with an unrelated class (e.g., “cat” for “bus”).

The experimental results reveal that VLP-SAM maintains a high level of performance across most semantic variations. Notably, configurations (b) through (g) all yield competitive results. High accuracy is maintained, even with ambiguous prompts such as “Hypernyms” and “Disambiguation,” demonstrating the model’s ability to handle broad semantic categories and multi-candidate scenarios. Furthermore, the strong performance achieved with “Descriptive Phrases” confirms that VLP-SAM captures the underlying semantic meaning of the text rather than merely memorizing fixed category names. The model also shows significant robustness to prompt formatting; for instance, the “Simple Label” (configuration (f)) achieves 75.7 mIoU, indicating that the segmentation accuracy remains stable regardless of the presence of the standard template. In contrast, performance significantly degrades when strong textual noise is introduced, such as in “Typos” (63.6 mIoU) and “Incorrect Label” (40.9 mIoU), where the linguistic structure is severely corrupted or misleading. These quantitative findings suggest that while VLP-SAM is highly robust to semantic shifts in language, it is sensitive to perturbations that disrupt the core linguistic structure. Overall, these results confirm that VLP-SAM is highly robust to a wide variety of textual formulations and formats while maintaining strong semantic generalization capability.

##### 5-Shot Testing

To further analyze the architecture, we evaluated VLP-SAM in a five-shot setting to examine the impact of increased reference information. We utilized five reference images as input to the VLP encode and averaged the resulting five prompt embeddings to generate a refined final prompt.

The results of the five-shot experiment are presented in Table 6 and Table 7, which show the performance on PASCAL-$5^{i}$ and COCO-$20^{i}$, respectively. VLP-SAM (CLIP Surgery) achieves higher performance than VRP-SAM (ResNet-50), outperforming it by 4.8% on PASCAL-$5^{i}$ and 5.3% on COCO-$20^{i}$.

However, when compared to the corresponding one-shot mIoU scores, the performance gain for VLP-SAM (CLIP Surgery) is only +0.1% on PASCAL-$5^{i}$ and −0.1% on COCO$20^{i}$. This observation suggests that the high-quality semantic information provided by the text label effectively saturates the required context at one-shot, leaving little room for further improvement through additional reference images. As analyzed in the following section, this saturation effect is primarily attributed to the efficiency of textual guidance, which effectively compensates for the incremental information gain typically provided by multiple reference images.

##### Impact of Prompt Quality on 5-Shot Performance

To further explore the information saturation effect of textual guidance and evaluate the model’s fusion capability, we conducted additional 5-shot experiments across various prompt qualities. Table 8 and Table 9 summarize the mIoU performance of VLP-SAM (CLIP Surgery) on PASCAL-$5^{i}$ and COCO-$20^{i}$ datasets under three prompt conditions: (1) “Incorrect” prompts, where the target category label is replaced by a completely unrelated class (e.g., replacing “horse” with “airplane”); (2) “Hypernym” prompts, which use a broad category label (e.g., “fruit” instead of “orange”); and (3) “Disambiguation” prompts, which provide multiple candidate labels (e.g., “[Target] or [Non-target]”).

In the “Incorrect” prompt setting, where the model must rely more on visual cues due to misleading textual noise, we observed a measurable gain from one-shot to five-shot (up to +0.5 mIoU on COCO-$20^{i}$). While this improvement is modest compared to visual-only models, it confirms that VLP-SAM possesses an inherent ability to fuse multiple reference images to compensate for unreliable textual guidance when necessary. Regarding vague descriptions such as “Hypernym” and “Disambiguation,” we observed that increasing the number of reference images to a 5-shot setting resulted in little to no performance improvement. However, it is important to note that even with these vague prompts, VLP-SAM achieves competitive accuracy at 1-shot that rivals the 5-shot performance of visual-only models like VRP-SAM. This indicates that the introduction of textual information effectively saturates the required semantic context at 1-shot, leaving little room for further improvement through additional reference images. In other words, the textual guidance is powerful enough to fully compensate for visual deficiencies, demonstrating high practical utility in real-world scenarios involving the collection of multiple annotated reference images is difficult.

##### Component Ablation

We evaluated the effectiveness of the main components in VLP-SAM through w/o experiments conducted on both PASCAL-$5^{i}$ and COCO-$20^{i}$ datasets. In this analysis, we investigated the contributions of the visual pseudo mask as well as the newly introduced textual prototype and attention mask. These components are designed to provide complementary spatial and semantic guidance to SAM for the few-shot segmentation task.

Table 10 and Table 11 present the results of these w/o experiments for PASCAL-$5^{i}$ and COCO-$20^{i}$, respectively. Configuration (a) represents the baseline without any prompt guidance, yielding 65.0 mIoU on PASCAL and 43.6 mIoU on COCO. Configuration (b) introduces the visual pseudo mask only, which provides a foundational performance gain of +3.4% and +5.3% over the baseline for PASCAL and COCO, respectively. Configuration (c) builds upon (b) by adding the textual prototype, resulting in further mIoU improvements to +4.7% and +10.0%. Finally, configuration (d), the full VLP-SAM model incorporating the attention mask, achieves the highest precision, with significant gains of +12.0% and +16.7% over the baseline.

Overall, these results clarify the distinct roles of each component: the visual pseudo mask establishes a spatial foundation, bringing the few-shot segmentation performance to a functional baseline level. The textual prototype then injects high-level semantic context to stabilize category identification. Most significantly, the attention mask acts as a semantic spatial mask that bridges the gap between conceptual and spatial information, leading to a substantial leap in precision. While the visual pseudo mask provides the necessary spatial grounding, the textual prototype and attention mask are the primary contributors to the model’s high accuracy, as they effectively resolve semantic ambiguities that visual initialization alone cannot address.

## 5. Discussion

In this section, we provide a detailed analysis of the effectiveness of VLP-SAM. The discussion is divided into two parts: (1) a qualitative analysis of visual segmentation results, and (2) a summary of the key architectural contributions and practical benefits of our proposed approach. This analysis aims to provide a clearer and more focused understanding of how VLP-SAM achieves its strong performance.

### 5.1. Qualitative Analysis of Visual Results

**Figure 4:**
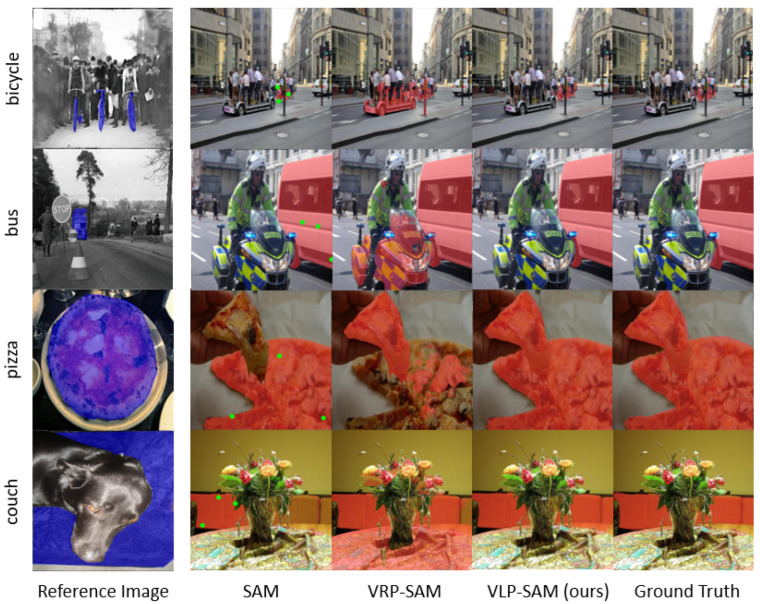
Visualization results of VLP-SAM compared to SAM and VRP-SAM on COCO-$20^{i}$ dataset for the bicycle, bus, pizza, and couch categories. VLP-SAM accurately predicts the target object by incorporating both a reference image and text label.

The proposed method exhibits a marked improvement over existing baselines by utilizing both reference images and text labels. This improvement is attributed to the integration of textual semantics, which facilitates the segmentation of complex objects such as “fully clothed people” or “visually similar but semantically distinct objects”. The qualitative improvements are highlighted in Figure 4, which demonstrate VLP-SAM’s ability to effectively distinguish semantically relevant objects.

Figure 4 illustrates comparative visualization results on the COCO-$20^{i}$ dataset for the categories bicycle, bus, pizza, and couch, none of which were seen during training. For baseline SAM, prompts are simulated by randomly sampling three positive points within the ground-truth mask. However, such prompts typically require per-image manual customization in real-world settings, significantly limiting the method’s usability. VRP-SAM, an existing few-shot method, uses ResNet-50 and only visual reference images. In contrast, our VLP-SAM is built upon CLIP Surgery and leverages both visual and textual information as multimodal prompts.

The visual comparisons reveal that VLP-SAM consistently achieves sharper and more semantically accurate segmentation compared to SAM and VRP-SAM. For example, in the category of bicycle, VRP-SAM segments the wrong objects based solely on the visual similarity of the reference image (e.g., the “tire” in the bicycle). VLP-SAM, on the other hand, accurately segments only the bicycle, guided by the semantic meaning conveyed by the text prompt “bicycle”. Similarly, in the category of bus, VRP-SAM segments a motorbike instead of a bus based solely on visual similarity, while VLP-SAM correctly segments the bus by leveraging textual semantics.

In the category of pizza, SAM and VRP-SAM partially fail to segment all pizza slices, while VLP-SAM successfully segments the full object set with high precision. The category of couch presents a challenging case where the reference image is ambiguous. VLP-SAM leverages the text prompt to segment the target region with improved accuracy. These examples collectively demonstrate VLP-SAM’s ability to incorporate semantic guidance via text in few-shot settings, enabling more accurate and context-aware segmentation compared to models relying solely on visual cues.

**Figure 5:**
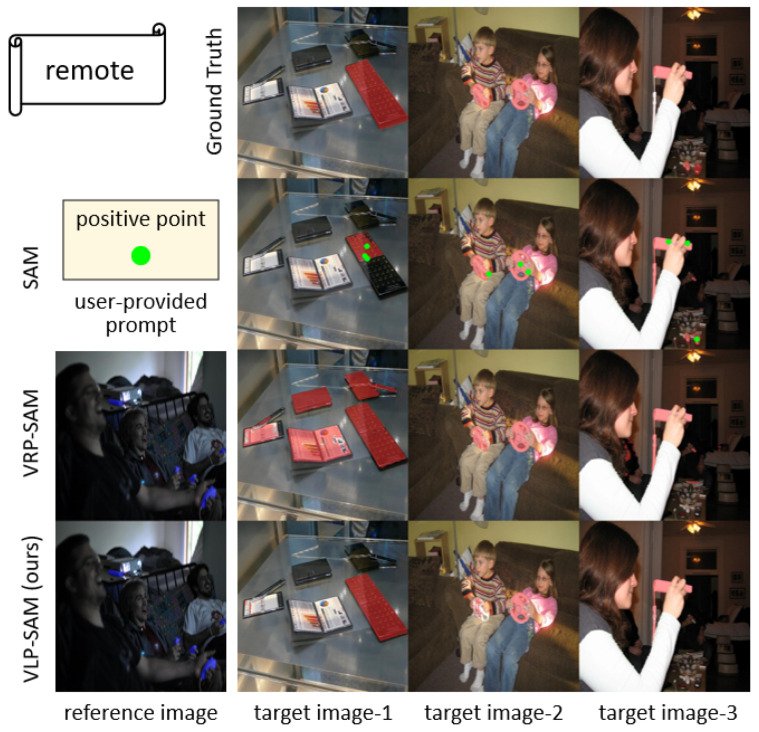
Visualization results of VLP-SAM compared to SAM and VRP-SAM on COCO-$20^{i}$ dataset for the “remote” category.

Furthermore, we examined cases where VLP-SAM faces challenges due to semantic misalignment between the target object and the textual label. As shown in Figure 5, target image-2 includes a Wii steering controller, which is a visually unique object that differs substantially from the generic concept of a “remote”. In this case, the text prompt fails to provide helpful semantic guidance, resulting in VLP-SAM misidentifying the object. Conversely, in target image-1, the same text prompt “remote” helps the model correctly segment a visually ambiguous object that reference images alone cannot resolve. These contrasting examples highlight that while VLP-SAM may underperform when the textual input does not adequately capture the semantics of the target object, the integration of textual modalities predominantly enhances segmentation quality in most scenarios. Additional visualization results across various scenarios, including a detailed analysis of failure cases under vague textual guidance, are provided in Appendix C to further validate the robustness and clarify the limitations of VLP-SAM.

### 5.2. Contribution of the Proposed Architecture

Beyond qualitative improvements, the design of VLP-SAM reflects a principled integration of multimodal components. Unlike SAM, which depends on carefully designed prompts such as points, boxes, or masks tailored to each individual image, and unlike VRP-SAM, which introduces reference images as a new input modality, VLP-SAM further extends the prompting space by integrating reference images with textual labels. This new framework not only enhances segmentation performance but also expands the potential of SAM-based architectures to support more flexible and multimodal prompting. The unified prompt embedding derived from visual and textual inputs enables robust performance without requiring manual, image-specific prompt engineering.

The scalability of VLP-SAM is further supported by its lightweight architecture, which introduces minimal learnable parameters while enabling strong generalization capabilities across diverse categories. Specifically, VLP-SAM contains only 1.4 M learnable parameters, significantly fewer than existing few-shot segmentation models such as CyCTR [61] (15.4 M), BAM [27] (4.9 M), and HDMNet [63] (4.2 M). Despite this efficiency, as shown in Table 3, VLP-SAM achieves superior performance on unseen classes in the COCO-$20^{i}$ dataset. Moreover, as shown in Table 6 and Table 7, the use of text information allows VLP-SAM to maintain high accuracy even with a single reference image. These results demonstrate that VLP-SAM offers both computational efficiency and practical effectiveness, making it well-suited for real-world few-shot segmentation tasks where annotated reference images are scarce.

Furthermore, the selection of CLIP Surgery as the backbone is motivated by its pixel–text matching capability, which is essential for segmentation tasks. Unlike standard CLIP, which focuses on global CLS tokens, CLIP Surgery captures local spatial semantics without requiring additional training. Notably, CLIP and CLIP Surgery are identical in terms of architecture, pretraining data, and model scale. Therefore, our method’s performance improvements are not due to increased model capacity, but rather to the careful integration of compatible architectures as shown in Table 1 and Table 2. Specifically, the introduction of the attention mask alongside the textual prototype yielded significant mIoU gains, as detailed in Table 10 and Table 11, which underscores the effectiveness of our proposed architectural components.

## 6. Conclusions and Future Work

In this paper, we proposed VLP-SAM, a novel SAM-based few-shot segmentation model that integrates both visual reference images and textual labels as prompts. VLP-SAM has a scalable and efficient architecture with only 1.4 M learnable parameters, utilizing a multimodal vision-language model to generate enriched prompt embeddings for SAM. By incorporating a newly designed prompt encoder, VLP-SAM achieves high accuracy and robust generalization. Comprehensive experiments on two benchmark datasets demonstrate that VLP-SAM significantly surpasses previous models by a large margin. Furthermore, VLP-SAM exhibits strong performance across unseen objects and cross-domain scenarios, highlighting its versatility for diverse real-world applications.

Future research will focus on further exploring the advantages of text-based prompting by evaluating performance across various text formats and under conditions involving noisy or imprecise textual inputs. We also intend to investigate the potential of integrating additional reference modalities, such as 3D data and depth maps, into the VLP encoder to leverage its simplicity and scalability. Conversely, by capitalizing on the extensive knowledge embedded within large-scale foundational models, we aim to reduce the reliance on annotated reference masks. This research direction holds the potential to enable segmentation with minimal supervision, eventually paving the way toward more flexible and open-vocabulary segmentation systems.

## Figures and Tables

**Table 1:** Performance of one-shot segmentation on PASCAL-$5^{i}$. Backbone indicates the image encoder within the novel SAM’s prompt encoder (VRP and VLP encoders). VLP-SAM outperforms the previous baseline VRP-SAM (ResNet-50) by a large margin (6.8% in mIoU). Bold font in the tables has been maintained to highlight the best performance for better readability.

Method	Backbone	Learnable Params	PASCAL-$5^{i}$				
			F-0	F-1	F-2	F-3	Mean
VRP-SAM	ResNet-50	1.6 M	72.2	75.5	69.0	63.9	70.2
	CLIP (ResNet-50)	1.5 M	64.4	70.4	64.4	57.7	64.3
	CLIP (ViT-B/16)	1.3 M	68.6	72.3	65.8	60.4	66.9
	CLIP Surgery (ViT-B/16)	1.3 M	70.5	75.9	65.4	61.6	68.4
VLP-SAM (ours)	CLIP (ViT-B/16)	1.4 M	71.9	73.1	65.3	62.2	68.1
	CLIP Surgery (ViT-B/16)	1.4 M	**76.9**	**83.1**	**72.3**	**75.6**	**77.0**

**Table 2:** Performance of one-shot segmentation on COCO-$20^{i}$. VLP-SAM outperforms the previous baseline VRP-SAM (ResNet-50) by a large margin (9.3% in mIoU). Bold font in the tables has been maintained to highlight the best performance for better readability.

Method	Backbone	Learnable Params	COCO-$20^{i}$				
			F-0	F-1	F-2	F-3	Mean
VRP-SAM	ResNet-50	1.6 M	44.3	55.5	53.5	50.8	51.0
	CLIP (ResNet-50)	1.5 M	30.5	41.5	43.7	40.1	38.9
	CLIP (ViT-B/16)	1.3 M	37.6	48.1	50.8	46.8	45.8
	CLIP Surgery (ViT-B/16)	1.3 M	40.6	52.3	52.8	49.9	48.9
VLP-SAM (ours)	CLIP (ViT-B/16)	1.4 M	41.7	52.8	52.5	51.7	49.7
	CLIP Surgery (ViT-B/16)	1.4 M	**52.8**	**63.7**	**63.2**	**61.6**	**60.3**

**Table 3:** Comparison with other few-shot segmentation models on COCO-$20^{i}$. These results are for one-shot segmentation. Backbone means the type of image encoder, and † denotes the reproduced version. VLP-SAM outperforms all previous models. Bold font in the tables has been maintained to highlight the best performance for better readability.

Method	Backbone	COCO-$20^{i}$				
		F-0	F-1	F-2	F-3	Mean
CyCTR	ResNet-50	38.9	43.0	39.6	39.8	40.3
BAM	ResNet-50	39.4	49.9	46.2	45.2	45.2
HDMNet	ResNet-50	43.8	55.3	51.6	49.4	50.0
PerSAM	SAM	23.1	23.6	22.0	23.4	23.0
PerSAM-F	SAM	22.3	24.0	23.4	24.1	23.5
Matcher	DINOv2	52.7	53.5	52.6	52.1	52.7
VRP-SAM †	ResNet-50	44.3	55.5	53.5	50.8	51.0
VRP-SAM †	CLIP Surgery (ViT-B/16)	40.6	52.3	52.8	49.9	48.9
VRP-SAM2	SAM2, ResNet-50	47.1	55.5	55.2	52.8	52.7
FCP	ResNet-50	46.4	56.4	55.3	51.8	52.5
CMaP-SAM	SAM	**54.0**	56.1	58.9	55.3	56.1
FS-SAM2	SAM2	49.3	57.6	58.3	56.1	55.3
VLP-SAM (ours)	CLIP (ViT-B/16)	41.7	52.8	52.5	51.7	49.7
	CLIP Surgery (ViT-B/16)	52.8	**63.7**	**63.2**	**61.6**	**60.3**

**Table 4:** Performance of one-shot segmentation under the cross-domain scenario from COCO-$20^{i}$ to PASCAL-$5^{i}$. VLP-SAM (CLIP Surgery) outperforms the previous text-free model, VRP-SAM (ResNet-50). Bold font in the tables has been maintained to highlight the best performance for better readability.

Method	Backbone	COCO-$20^{i}$ → PASCAL-$5^{i}$				
		F-0	F-1	F-2	F-3	Mean
VRP-SAM	ResNet-50	**63.6**	77.3	78.3	86.8	76.5
	CLIP (ResNet-50)	51.1	72.8	65.2	82.6	67.9
	CLIP (ViT-B/16)	57.0	76.0	73.3	85.2	72.9
	CLIP Surgery (ViT-B/16)	55.5	79.2	75.8	87.3	74.5
VLP-SAM (ours)	CLIP (ViT-B/16)	57.2	79.2	74.3	85.3	74.0
	CLIP Surgery (ViT-B/16)	61.8	**80.4**	**82.9**	**91.9**	**79.3**

**Table 5:** Robustness analysis of VLP-SAM (CLIP Surgery) across diverse textual prompt variations on PASCAL-$5^{i}$ in the 1-shot setting. Configuration (a) is the original template used in other experiments.

	Prompt Type	F-0	F-1	F-2	F-3	Mean
(a)	Original (a photo of a [label])	76.9	83.1	72.3	75.6	77.0
(b)	Synonyms	69.1	81.7	72.6	67.6	72.8
(c)	Hypernyms	67.9	79.3	73.1	73.4	73.4
(d)	Disambiguation (A or B)	73.8	80.5	70.7	72.4	74.4
(e)	Descriptive Phrases	72.9	78.6	71.5	76.6	74.9
(f)	Simple Label	75.2	82.0	72.5	73.1	75.7
(g)	Label Description	75.7	81.4	72.7	75.7	76.4
(h)	Typos	60.7	69.7	62.0	62.1	63.6
(i)	Incorrect Label	30.2	61.8	45.0	26.6	40.9

**Table 6:** Performance of 5-shot segmentation on PASCAL-$5^{i}$. The values of (+x.x) in Mean indicate the performance gain compared to the one-shot setting. Bold font in the tables has been maintained to highlight the best performance for better readability.

Method	Backbone	PASCAL-$5^{i}$				
		F-0	F-1	F-2	F-3	Mean
VRP-SAM	ResNet-50	75.2	76.8	71.1	66.0	72.3 (+2.1)
	CLIP (ResNet-50)	60.4	72.0	64.8	59.7	64.2 (−0.1)
	CLIP (ViT-B/16)	70.4	73.8	66.5	60.9	67.9 (+1.0)
	CLIP Surgery (ViT-B/16)	72.7	77.0	66.5	62.6	69.7 (+1.3)
VLP-SAM (ours)	CLIP (ViT-B/16)	72.0	73.1	65.3	62.3	68.2 (+0.1)
	CLIP Surgery (ViT-B/16)	**76.9**	**83.2**	**72.8**	**75.4**	**77.1** (+0.1)

**Table 7:** Performance of 5-shot segmentation on COCO-$20^{i}$. The values of (+x.x) in Mean indicate the performance gain compared to the one-shot setting. Bold font in the tables has been maintained to highlight the best performance for better readability.

Method	Backbone	COCO-$20^{i}$				
		F-0	F-1	F-2	F-3	Mean
VRP-SAM	ResNet-50	47.7	58.2	57.1	56.5	54.9 (+3.9)
	CLIP (ResNet-50)	33.3	44.5	45.1	44.3	41.8 (+2.9)
	CLIP (ViT-B/16)	41.6	52.0	52.6	52.9	49.8 (+4.0)
	CLIP Surgery (ViT-B/16)	45.8	57.3	55.2	56.6	53.7 (+4.8)
VLP-SAM (ours)	CLIP (ViT-B/16)	42.2	54.0	53.7	52.1	50.5 (+0.8)
	CLIP Surgery (ViT-B/16)	**52.4**	**62.7**	**63.4**	**62.2**	**60.2** (−0.1)

**Table 8:** Performance of 5-shot segmentation using VLP-SAM (CLIP Surgery) with varying textual prompt quality on PASCAL-$5^{i}$. The values of (+x.x) in Mean indicate the performance gain compared to the one-shot setting.

Prompt Type	PASCAL-$5^{i}$				
	F-0	F-1	F-2	F-3	Mean
Original (a photo of a [label])	76.9	83.2	72.8	75.4	77.1 (+0.1)
Disambiguation	73.7	80.6	70.7	72.3	74.3 (+0.0)
Hypernym	67.6	79.4	73.1	73.4	73.4 (+0.0)
Incorrect	30.4	61.9	45.3	26.6	41.1 (+0.2)

**Table 9:** Performance of 5-shot segmentation using VLP-SAM (CLIP Surgery) with varying textual prompt quality on COCO-$20^{i}$. The values of (+x.x) in Mean indicate the performance gain compared to the one-shot setting.

Prompt Type	COCO-$20^{i}$				
	F-0	F-1	F-2	F-3	Mean
Original (a photo of a [label])	52.4	62.7	63.4	62.2	60.2 (−0.1)
Disambiguation	45.8	58.2	61.6	57.9	55.9 (−0.4)
Hypernym	45.1	55.3	58.2	54.9	53.4 (−0.3)
Incorrect	22.9	23.8	35.2	25.2	26.8 (+0.5)

**Table 10:** Ablation study for different components of VLP-SAM (CLIP Surgery) on PASCAL-$5^{i}$. Configuration (a) represents the baseline without any prompt guidance, and the values of **Δ** indicate the performance gain compared to configuration (a).

	Pseudo	Textual	Attention	PASCAL-$5^{i}$					
	Mask	Prototype	Mask	F-0	F-1	F-2	F-3	Mean	Δ
(a)				65.6	73.0	65.0	56.4	65.0	0.0
(b)	✓			70.5	75.9	65.4	61.6	68.4	+3.4
(c)	✓	✓		74.7	75.4	65.3	63.2	69.7	+4.7
(d)	✓	✓	✓	76.9	83.1	72.3	75.6	77.0	+12.0

**Table 11:** Ablation study for different components of VLP-SAM (CLIP Surgery) on COCO-$20^{i}$. Configuration (a) represents the baseline without any prompt guidance, and the values of **Δ** indicate the performance gain compared to configuration (a).

	Pseudo	Textual	Attention	COCO-$20^{i}$					
	Mask	Prototype	Mask	F-0	F-1	F-2	F-3	Mean	Δ
(a)				36.6	48.6	44.9	44.2	43.6	0.0
(b)	✓			40.6	52.3	52.8	49.9	48.9	+5.3
(c)	✓	✓		46.5	58.5	55.5	53.7	53.6	+10.0
(d)	✓	✓	✓	52.8	63.7	63.2	61.6	60.3	+16.7

**Figure A1:**
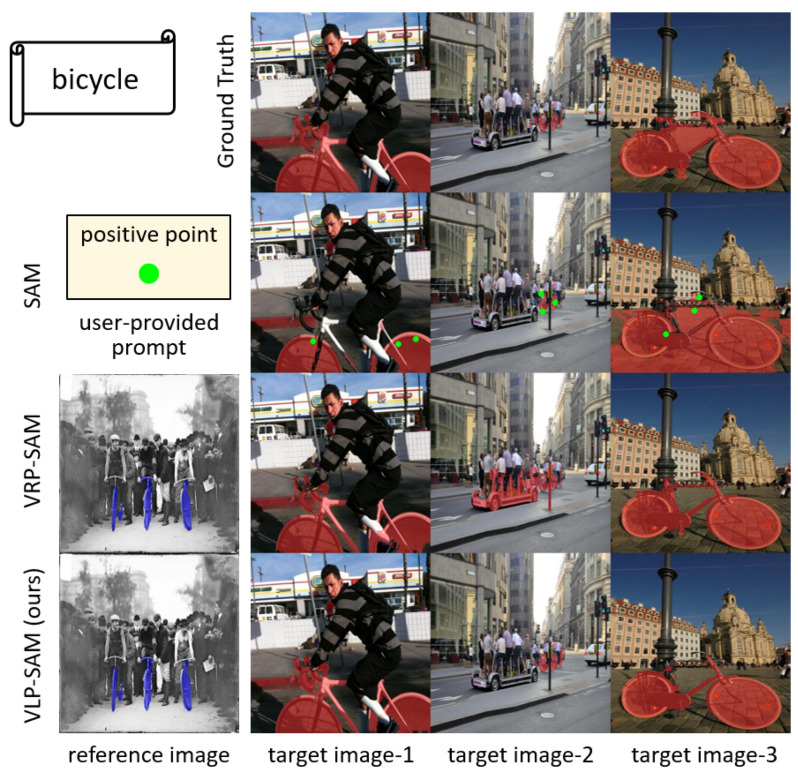
Visualization results for the bicycle category. VLP-SAM utilizes both the annotated reference image and text label to effectively localize the target object without manual prompt engineering.

**Figure A2:**
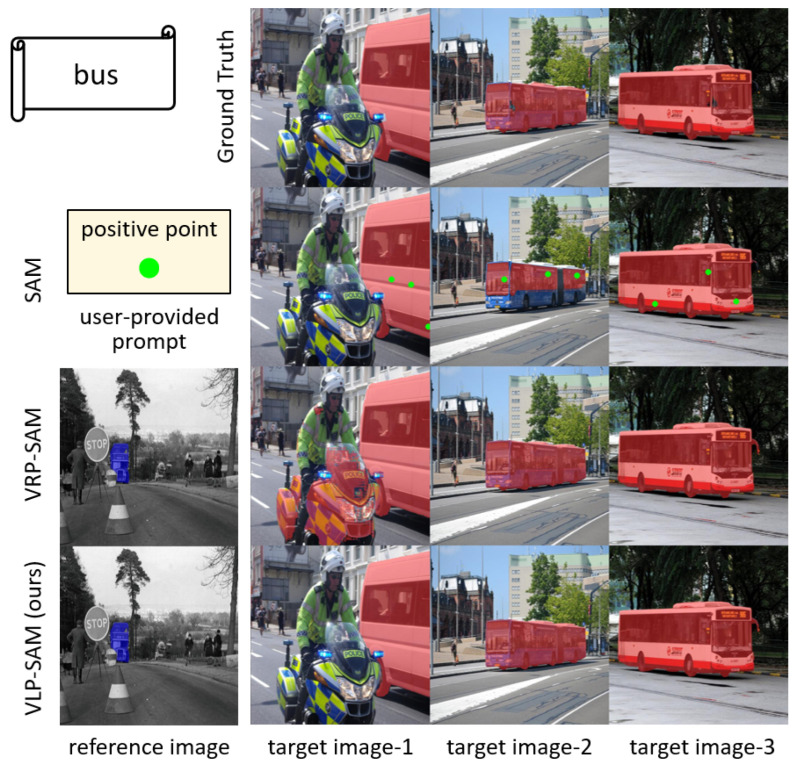
Visualization results for the bus category. By incorporating multimodal prompts, VLP-SAM accurately distinguishes the bus from visually similar vehicles.

**Figure A3:**
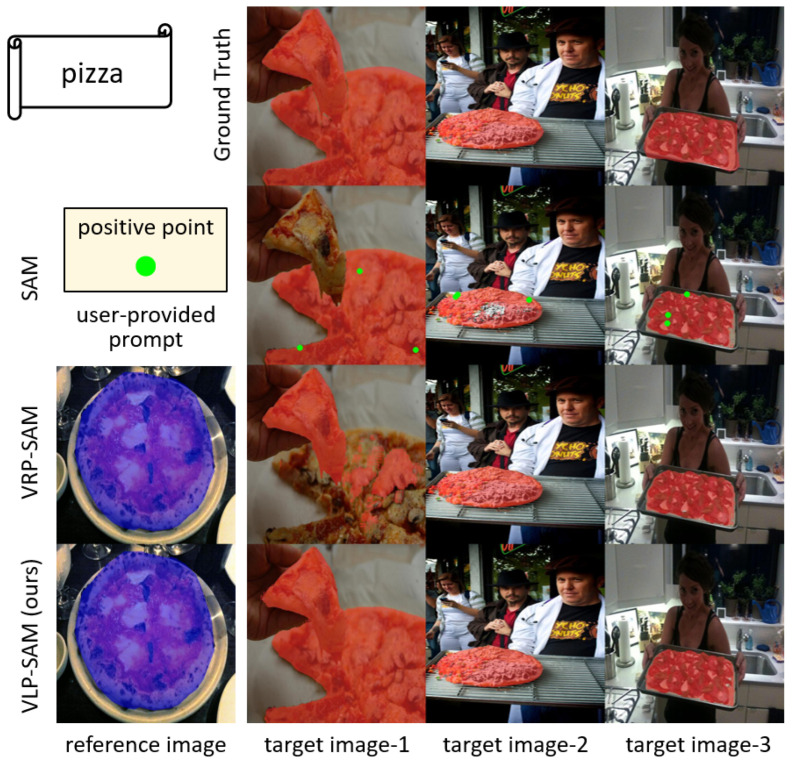
Visualization results for the pizza category. VLP-SAM demonstrates superior part-to-whole understanding, successfully segmenting all slices with high precision.

**Figure A4:**
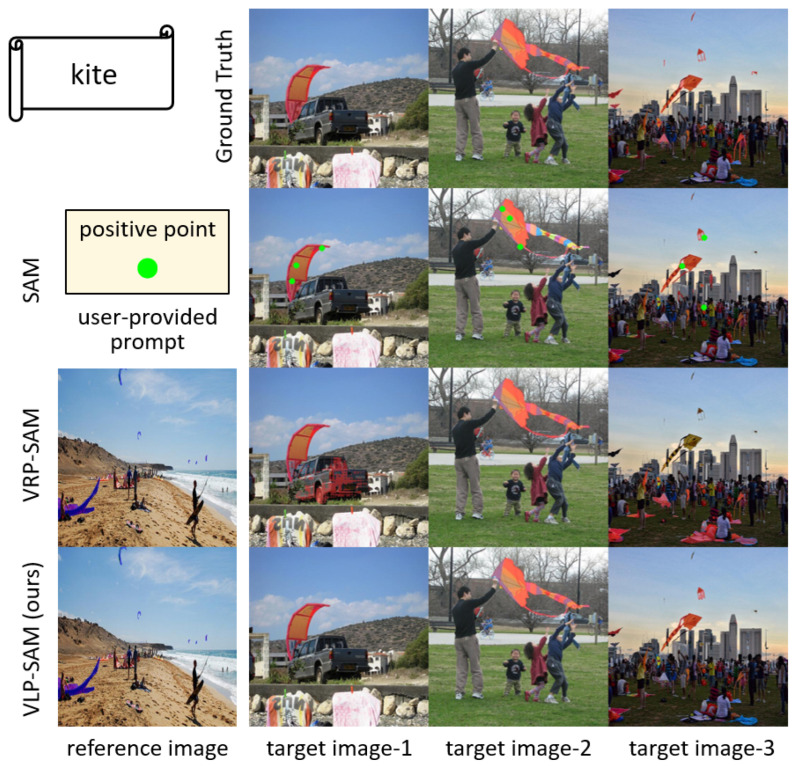
Visualization results for the kite category. The semantic guidance provided by the text label enables accurate segmentation of thin structures and complex patterns.

**Figure A5:**
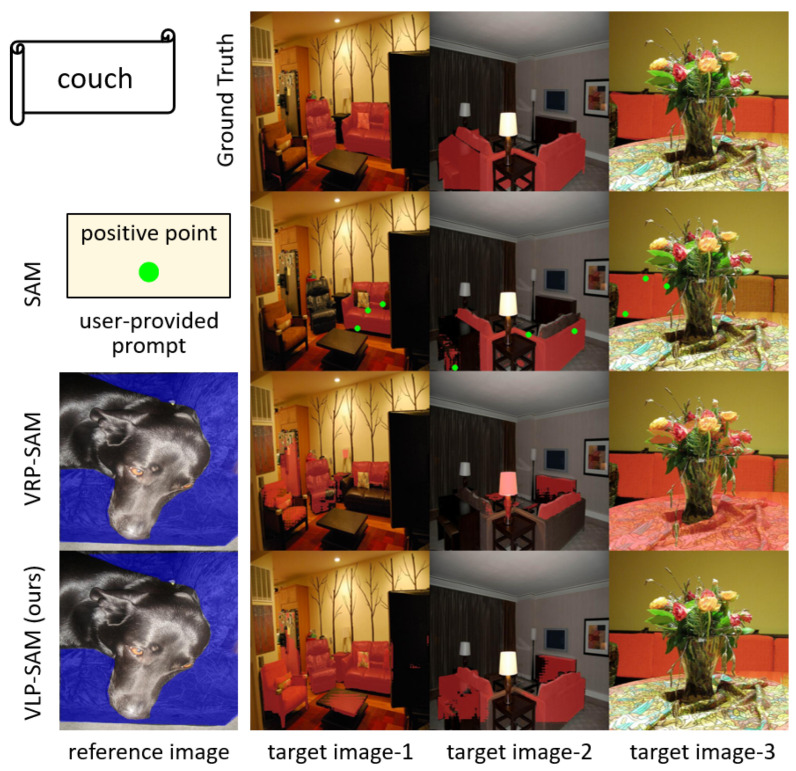
Visualization results for the couch category. VLP-SAM effectively leverages textual semantics to resolve ambiguities when the reference visual features are insufficient.

**Figure A6:**
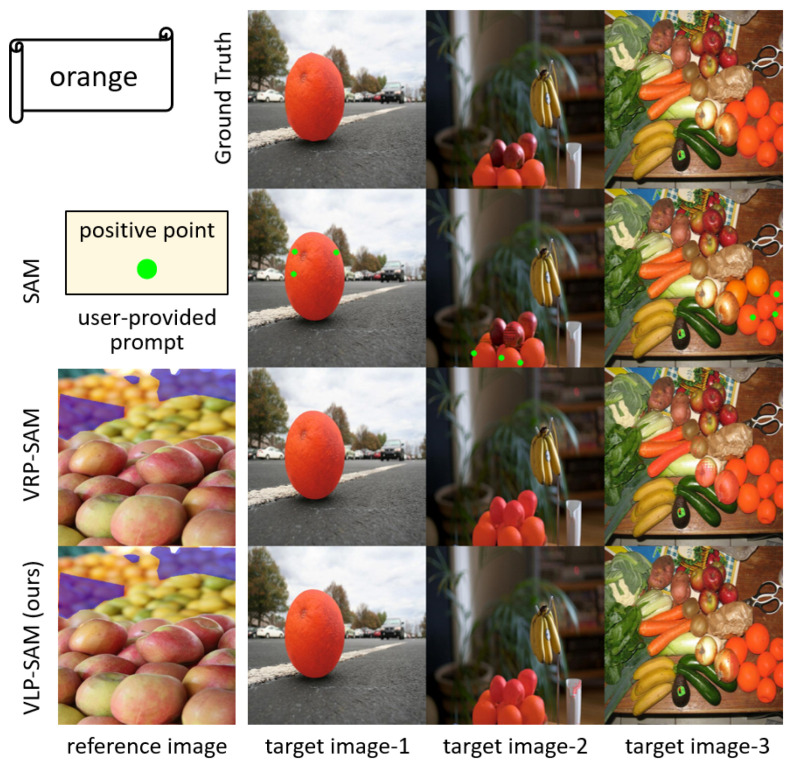
Visualization results for the orange category. The model successfully segments the target objects in a cluttered environment by aligning visual features with the category label.

**Figure A7:**
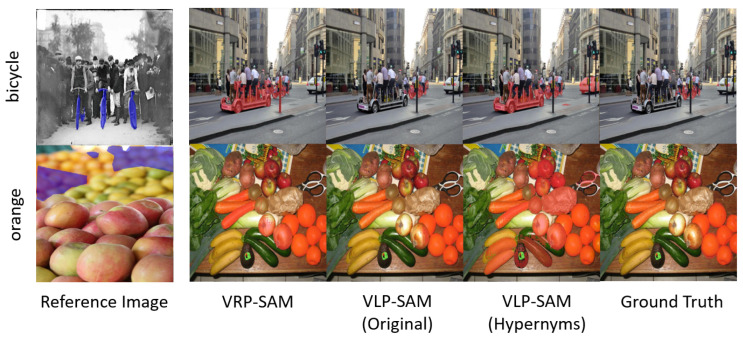
Failure case analysis with hypernym prompts on COCO-$20^{i}$. From left to right: reference image, VRP-SAM, VLP-SAM (original), VLP-SAM (hypernyms), and ground truth. In the first line (bicycle), the hypernym “non-motorized vehicle” causes over-segmentation of non-target vehicles. In the second line (orange), the hypernym “fruit” leads to the erroneous segmentation of other fruit types.

## Data Availability

The data presented in this study are openly available in the public domain. The PASCAL-5^i^ dataset was derived from the PASCAL VOC 2012 repository (http://host.robots.ox.ac.uk/pascal/VOC/voc2012/), accessed on 20 March 2026. The COCO-20^i^ dataset was derived from the MS COCO 2014 repository (https://cocodataset.org/), accessed on 20 March 2026. The code developed in this study are available at https://github.com/kosukesakurai1/VLP-SAM, accessed on 20 March 2026.

## Appendix A. Detailed Data Splits for Evaluation

This section provides the comprehensive classification of object categories used within the respective test subsets. Table A1 and Table A2 detail the testing classes for PASCAL-$5^{i}$ and COCO-$20^{i}$, respectively. Additionally, Table A3 specifies the class alignment used for the cross-domain scenario between PASCAL-$5^{i}$ and COCO-$20^{i}$.

**Table A1:** Details of the data split for PASCAL-$5^{i}$ [8]. Each row consists of 5 classes designated for testing, with the remaining 15 classes utilized for training.

Fold	Test Classes
0	Aeroplane, Bicycle, Bird, Boat, Bottle
1	Bus, Car, Cat, Chair, Cow
2	Dining table, Dog, Horse, Motorbike, Person
3	Potted plant, Sheep, Sofa, Train, TV/monitor

**Table A2:** Details of the data split for COCO-$20^{i}$ [9]. Each row consists of 20 classes designated for testing, with the remaining 60 classes utilized for training.

Fold	Test Classes
0	Person, Airplane, Boat, Parking meter, Dog, Elephant, Backpack,
	Suitcase, Sports ball, Skateboard, Wine glass, Spoon, Sandwich,
	Hot dog, Chair, Dining table, Mouse, Microwave, Sink, Scissors
1	Bicycle, Bus, Traffic light, Bench, Horse, Bear, Umbrella, Frisbee,
	Kite, Surfboard, Cup, Bowl, Spoon, Orange, Pizza, Couch, Toilet,
	Remote, Oven, Book, Teddy bear
2	Car, Train, Fire hydrant, Bird, Sheep, Zebra, Handbag, Skis,
	Baseball bat, Tennis racket, Fork, Banana, Broccoli, Donut,
	Potted plant, TV, Keyboard, Toaster, Clock, Hair drier
3	Motorcycle, Truck, Stop sign, Cat, Cow, Giraffe, Tie, Snowboard,
	Baseball glove, Bottle, Knife, Apple, Carrot, Cake, Bed, Laptop,
	Cell phone, Sink, Vase, Toothbrush

**Table A3:** Details of the data split for PASCAL-$5^{i}$ in cross-domain scenario. Each line represents non-overlapping classes in the training set corresponding to the respective fold of COCO-$20^{i}$.

Fold	Test Classes
0	Aeroplane, Boat, Chair, Dining table, Dog, Person
1	Horse, Sofa, Bicycle, Bus
2	Bird, Car, Potted plant, Sheep, Train, TV/monitor
3	Bottle, Cow, Cat, Motorbike

## Appendix B. Detailed Statistical Analysis

To evaluate the empirical validity and stability of VLP-SAM, we conducted a rigorous statistical analysis across five random seeds by varying the selection of reference images for each run. While the main manuscript reports the mean mIoU values, Table A4 provides the detailed performance, including standard deviations (SD) for both PASCAL-$5^{i}$ and COCO-$20^{i}$ datasets.

**Table A4:** Detailed statistical performance comparison (mean mIoU ± SD) on PASCAL-$5^{i}$ and COCO-$20^{i}$ across 5 random seeds in the 1-shot setting. Bold font in the tables has been maintained to highlight the best performance for better readability.

Dataset	Method	F-0	F-1	F-2	F-3	Mean
PASCAL-$5^{i}$	VRP-SAM (ResNet)	72.2±0.7	75.5±0.5	69.0±0.6	63.9±0.4	70.2±0.5
	VLP-SAM (Ours)	76.9±0.1	83.1±0.4	72.3±0.3	75.6±0.2	77.0±0.2
COCO-$20^{i}$	VRP-SAM (ResNet)	44.3±1.1	55.5±2.3	53.5±1.5	50.8±1.7	51.0±1.7
	VLP-SAM (Ours)	52.8±1.8	63.7±1.3	63.2±1.6	61.6±1.5	60.3±1.5

We compared our VLP-SAM (CLIP Surgery) with the previous visual-only model, VRP-SAM (ResNet-50). The results confirm that VLP-SAM consistently outperforms the baseline across all folds with narrow standard deviations. Specifically, in the challenging COCO-$20^{i}$ dataset, where background clutter often increases variability, VLP-SAM maintains a stable performance lead. These performance gains are statistically significant, as the margins of improvement consistently exceed the standard deviations. This confirms that the results achieved by incorporating textual guidance are statistically robust and not a result of random initialization.

## Appendix C. Additional Qualitative Results

Figure A1–Figure A6 provide a comprehensive set of comparative visualization results on the COCO-$20^{i}$ dataset for categories including bicycle, bus, pizza, kite, couch, and orange. Notably, all these categories were unseen during the training phase. These results further validate that VLP-SAM consistently achieves more precise and semantically accurate segmentation than SAM and VRP-SAM across a wide range of object scales and complexities.

Furthermore, Figure A7 illustrates specific failure cases when utilizing extremely vague textual prompts (Hypernyms) to explore the model’s limitations. For the bicycle category, when provided with the hypernym “non-motorized vehicle,” VLP-SAM (Hypernyms) fails to maintain class specificity and erroneously segments other nearby vehicles that belong to the same broad category, whereas VLP-SAM (Original) correctly identifies only the target bicycles. Similarly, for the orange category, using the hypernym “fruit” leads the model to over-segment other fruit instances, such as bananas and apples, present in the background. These cases indicate that while VLP-SAM possesses strong semantic understanding, its precision is inherently tied to the specificity of the textual guidance; overly broad descriptions can lead to semantic expansion beyond the intended target.
